# A commentary on TREAT: The trial of early aggressive drug therapy in juvenile idiopathic arthritis

**DOI:** 10.1186/1741-7015-10-59

**Published:** 2012-06-13

**Authors:** Eileen Baildam

**Affiliations:** 1 Department of Paediatric Rheumatology, Alder Hey Children's Foundation NHS Trust, Liverpool, UK

**Keywords:** Childhood arthritis, polyarticular juvenile arthritis, treatment

## Abstract

Polyarticular juvenile idiopathic arthritis (JIA) is a category of JIA where multiple joints are affected by chronic inflammation, and where serious and lasting damage to joints is the expected natural history in untreated disease. There is evidence of response to disease-modifying antirheumatic and biologic drugs, but little evidence of permanent remission from any of the existing therapeutic trials. The TREAT trial by Wallace *et al.*, recently published in *Arthritis and Rheumatism*, used a collaborative multicenter approach to studying early aggressive treatment of polyarticular JIA in an attempt to achieve full clinical inactive disease after 6 months of treatment. The study's main finding that the earlier in the disease course that treatment is started, the better the chance of disease control, has provided evidence that there is a 'window of opportunity' for treating JIA as there is in adult rheumatoid arthritis (RA). The study provides both a platform and an impetus for concentrating future treatment trials on early rather than established disease and investigating a standard of starting treatment within 10 to 12 weeks.

## Background

The term juvenile idiopathic arthritis (JIA) is used to cover a group of conditions in childhood from infancy to the age of 17 where arthritis is the main feature and is truly idiopathic (that is, where no definite infective, malignant, hematological or other autoimmune cause is found for the arthritis). There are various subtypes defined in the JIA spectrum, agreed by expert consensus (International League of Associations for Rheumatology (ILAR) criteria) [1,2] to be as near to individual disease entities as possible, especially for the purpose of future research into more exact etiologies for these conditions.

Polyarticular JIA is defined as the JIA type where five or more joints are involved with either a rheumatoid factor-positive (on two consecutive tests) or a rheumatoid factor-negative further subdivision [1,2]. JIA is said to be polyarticular if at any time in the first 6 months from onset the cumulative number of involved joints reaches five. If this additive total effect only occurs after 6 months from onset then the type is changed from oligoarticular (up to four joints involved) to extended oligoarticular (five or more joints after 6 months). In addition, the JIA subtypes of systemic onset JIA and enthesitis related JIA, as well as psoriatic JIA, can also involve five or more joints. If the other associated defining features of any these particular groups are missing or overlooked, it is possible for a clinical case to be assigned to the incorrect group. Indeed, there may be some false divisions between the various subtypes if polyarticular arthritis is the main feature. However, there are still too few studies with large enough subgroups of patients with all the subtypes to know if the ILAR classification criteria for subtypes is truly clinically relevant from a therapeutic point of view in disease with polyarticular involvement. There is no single diagnostic test for any of the subtypes of JIA apart from a persistently raised rheumatoid factor level in the absence of connective tissues disorders.

The incidence of JIA is approximately 1:10,000, with a prevalence in the population of around 1:1,000 and about one-third of cases being polyarticular in nature [3,4]. This subtype may affect from five to most joints, can cause erosive disease, joint deformities with damage to growth plates, reduced mobility and general debility from chronic inflammation. It is associated with long-term disease activity into adult life in over 60% of cases. Treatment to achieve clinical remission is therefore vital, but the exact algorithm of care to achieve full remission has still not been established and there is considerable variation between centers based on clinical experience.

The role of methotrexate in the polyarticular subtype is secure after controlled trials in relatively large numbers of patients [5-7]. Biologic therapies including anti-tumor necrosis factor (TNF) blockers have been shown to improve disease activity, but all treatment studies seem to plateau with 75% to 85% of patients responding at significant response levels [8]. Steroids are used in different ways, with short-term intravenous courses of methylprednisolone or one-off intramuscular depot injections and/or multiple and repeated intra-articular injections being favored by some with others using more long-term regimes of low dose oral steroids [9-11]. For difficult unresponsive and damaging disease there are suggestions that Rituximab use may have an important role [12]. So far, complete and sustained clinical remission allowing for complete withdrawal of treatment is rarely reported, and yet this remains the long-term aim of all treatment attempts [13-16].

## The TREAT trial: main findings and strengths of the study design

There have been no studies in JIA designed to investigate an initial aggressive approach to treatment with an aim of early complete disease remission, although many clinicians individualize treatments in an effort to achieve this. The TREAT trial [17] compared subcutaneous methotrexate at 0.5 mg/kg/week to a maximum of 40 mg plus two placebos with the same methotrexate regime plus subcutaneous etanercept 0.8 mg/kg/week (maximum 50 mg) and oral prednisolone 0.5 mg/kg/day (maximum 60 mg) tapered to 0 mg by 17 weeks.

The ambitious primary outcome was set as clinical inactive disease [18] at 6 months with clinical remission on medication within 12 months of treatment also assessed (defined as a continuous 6 months of clinically inactive disease while receiving medication).

The strength of this study was that it attempted to use the 'window of opportunity' approach for long-lasting disease control from aggressive initial treatment [13]. Rather than simply looking for improvement using the usual American College of Rheumatology (ACR) Paediatric 70 response rates (70% improvement) the much more stringent primary outcome of clinically inactive disease was used. Assessment of two active treatments versus two concurrent placebos was also a strength, as it involved analyzing a treatment algorithm rather than looking at a single drug effect.

The main finding of the study was that after 12 months clinical remission on treatment was achieved in significantly more patients in the intensive treatment group than in the placebo and methotrexate group (*P *= 0.0534).

Potentially the most important finding of the study was that the shorter the disease duration was at baseline, the more likely it was that clinically inactive disease would be achieved by 6 months. For each month sooner after disease onset the aggressive treatment was begun, the more likely it was that clinical remission at 6 months would be achieved, and this was to a factor of 1.324 per month earlier. This finding does support the very important suggestion of a 'window of opportunity' for easier and more long-lasting disease control [13].

## Limitations of the TREAT trial

There were disappointingly small numbers of patients achieving clinical remission on treatment by 12 months with 9/42 (21%) in the full treatment arm and 3/43 (7%) in the methotrexate and double placebo arm, suggesting that although this difference was statistically significant the treatment regime itself is unlikely to be the breakthrough in terms of the majority of patients that we are seeking. However, these patients had very severe disease and a large proportion (33% to 40%) had rheumatoid factor positivity.

The randomization process did allocate more severe patients to the placebo arm in terms of more active joints and a significantly higher erythrocyte sedimentation rate (ESR) in the placebo arm, but this should really have served to accentuate the effect of the full treatment regime. This study demonstrated how effective injected weekly methotrexate can be given early in the disease course.

The relatively large numbers of patients discontinuing complex studies such as this provides a warning to investigators to include larger numbers of patients than the strict power calculations allow in order that the final numbers in each group remain meaningful. Larger numbers also allow for more subanalyses to be carried out in the explanation of results.

It is possible that once-weekly etanercept is not as effective as twice-weekly etanercept in the initial phases of treatment and that oral prednisolone is not as effective as intramuscular, intravenous or multiple intra-articular routes of steroid administration. The definition of clinically inactive disease may have been too stringent in this study with the 2011 consensus for provisional criteria for definitions containing a few amendments that may be more realistic [19].

## Conclusions

These findings support the need to continue the search for a rapid initial remission-inducing regime, and yet the protocol studied does not appear to be that exact regime. The findings that the earlier treatment is started the more likely that clinical remission on treatment will be achieved at 12 months is a fundamental one in encouraging clinicians and healthcare providers to continue to be open to new regimes, rather than enshrining accepted protocols such as National Institute for Health and Clinical Excellence (NICE)-agreed protocols in practice forever.

The need now is for a study of 'tight control' until clinical inactive disease is achieved using a personalized approach, with addition of therapies with short interval reviews until the patient is fully under control (as has been shown in adult rheumatoid arthritis), and then to describe what these regimes require [19-22]. The results of the TREAT trial are worrying enough to justify more substantial treatments such as a trial of rituximab on first diagnosis of polyarticular JIA rather than after failure of more widely used treatments.

## Competing interests

The author declares that they have no competing interests.

## Author information

EB is Consultant Paediatric Rheumatologist at Alder Hey Children's Foundation NHS Trust and Deputy Chair of the Clinical Studies Group in Paediatric Rheumatology, a part of the UK Medicines for Children Research Network and Arthritis Research UK

## Pre-publication history

The pre-publication history for this paper can be accessed here:

http://www.biomedcentral.com/1741-7015/10/59/prepub
